# Supplementary material for: Deficient humoral responses and disrupted B-cell immunity are associated with fatal SFTSV infection
Source: Nat Commun. 2018 Aug 20;9:3328. doi: 10.1038/s41467-018-05746-9 (PMC6102208; doi:10.1038/s41467-018-05746-9)
Supplement: Supplementary file 1 — Supplementary Information [file 41467_2018_5746_MOESM1_ESM.pdf]

Deficient humoral responses and disrupted B cell immunity are associated with fatal SFTSV infection

Song et al

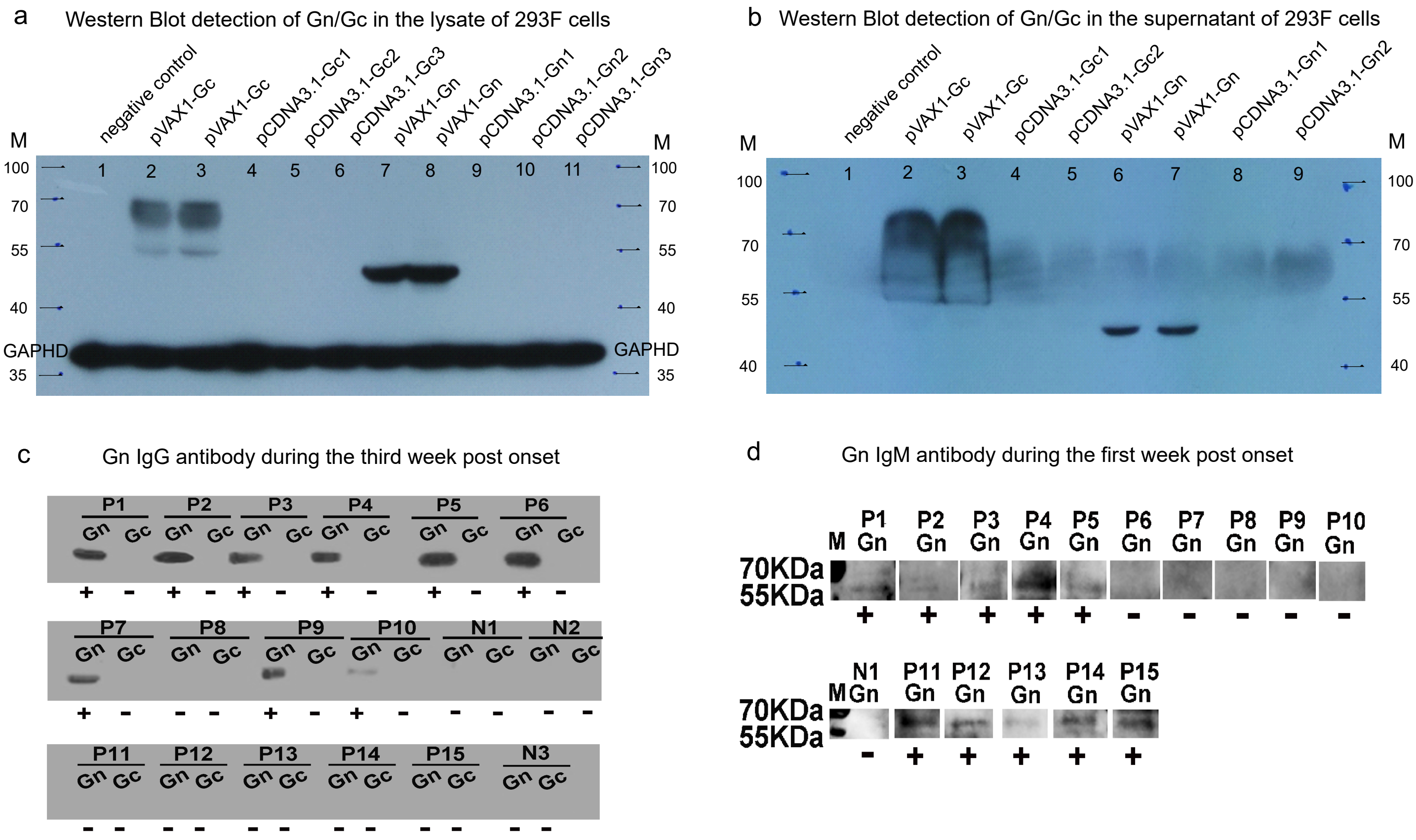

Supplementary Fig. 1 Identification of Gn/Gc and detection of Gn specific IgG/IgM in patients' sera . The expression of Gn and Gc in cell lysate (a) and in the supernatant (b) of 293F cells. GAPDH served as the internal control and M denotes the molecular marker. (c) Western blot analysis of serum antibodies to Gn and Gc of 10 survived (P1-P10) and 5 deceased (P11-P15) patients during the 3rd week post symptom onset. The experiment was performed three times and the representative image was presented. N1-N3 represent three healthy individuals. (d) Western blot detection of SFTSV Gn specific IgM in patients' sera during the 1st week post symptom onset. P1-P10 and P11-P15 represent ten survived and five deceased patients, respectively. M denotes the molecular marker indicating Gn detected by serum IgM. "+" and "-" denote positive and negative detections, respectively.

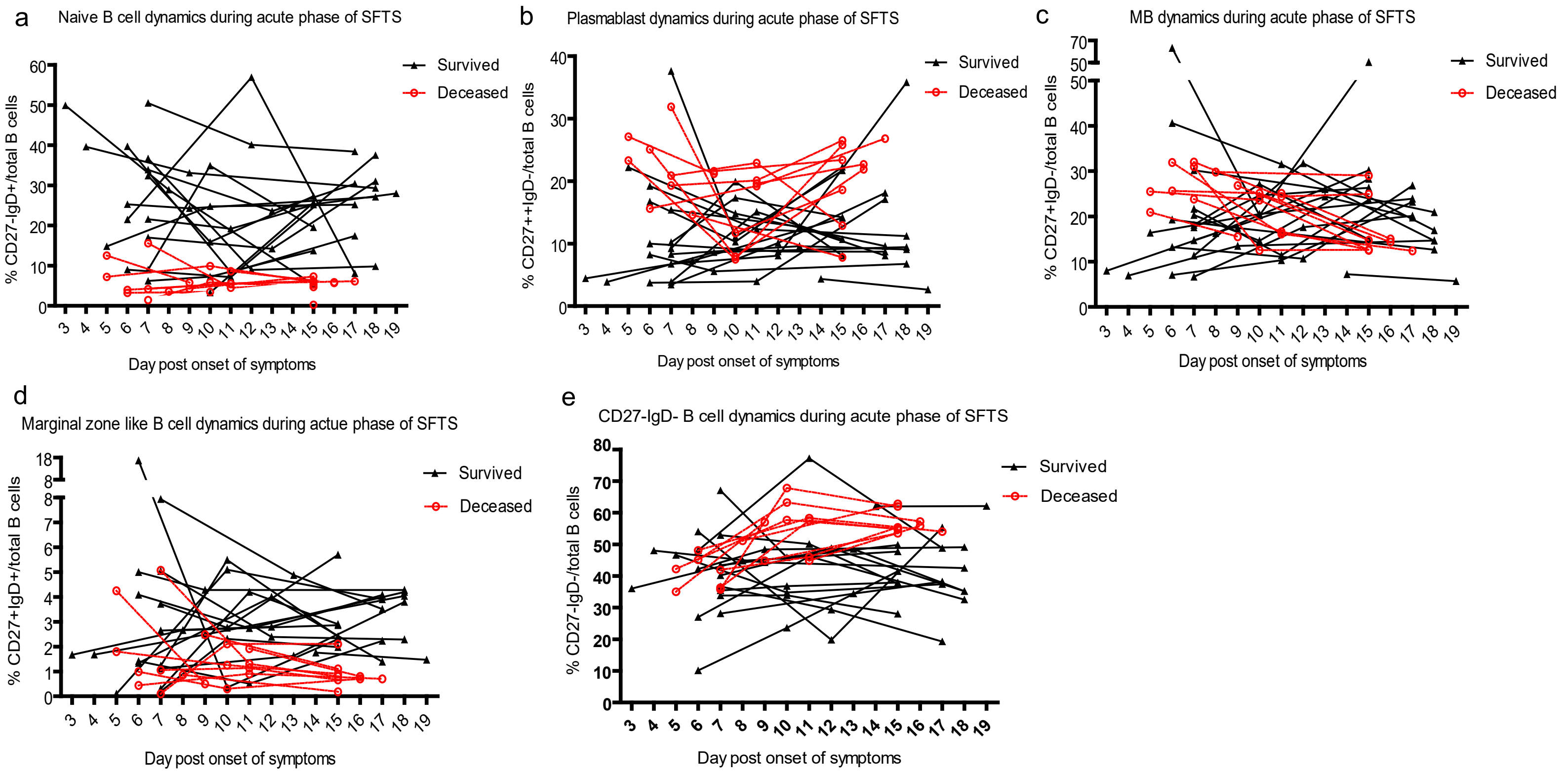

Supplementary Fig. 2 Dynamics of peripheral B cell subsets in SFTS patients during acute infection. The percentages of major B cell subsets in total peripheral B cells of 17 survived and 10 deceased patients over the entire three-week clinical course post symptom onset. Each line represents an individual patient.

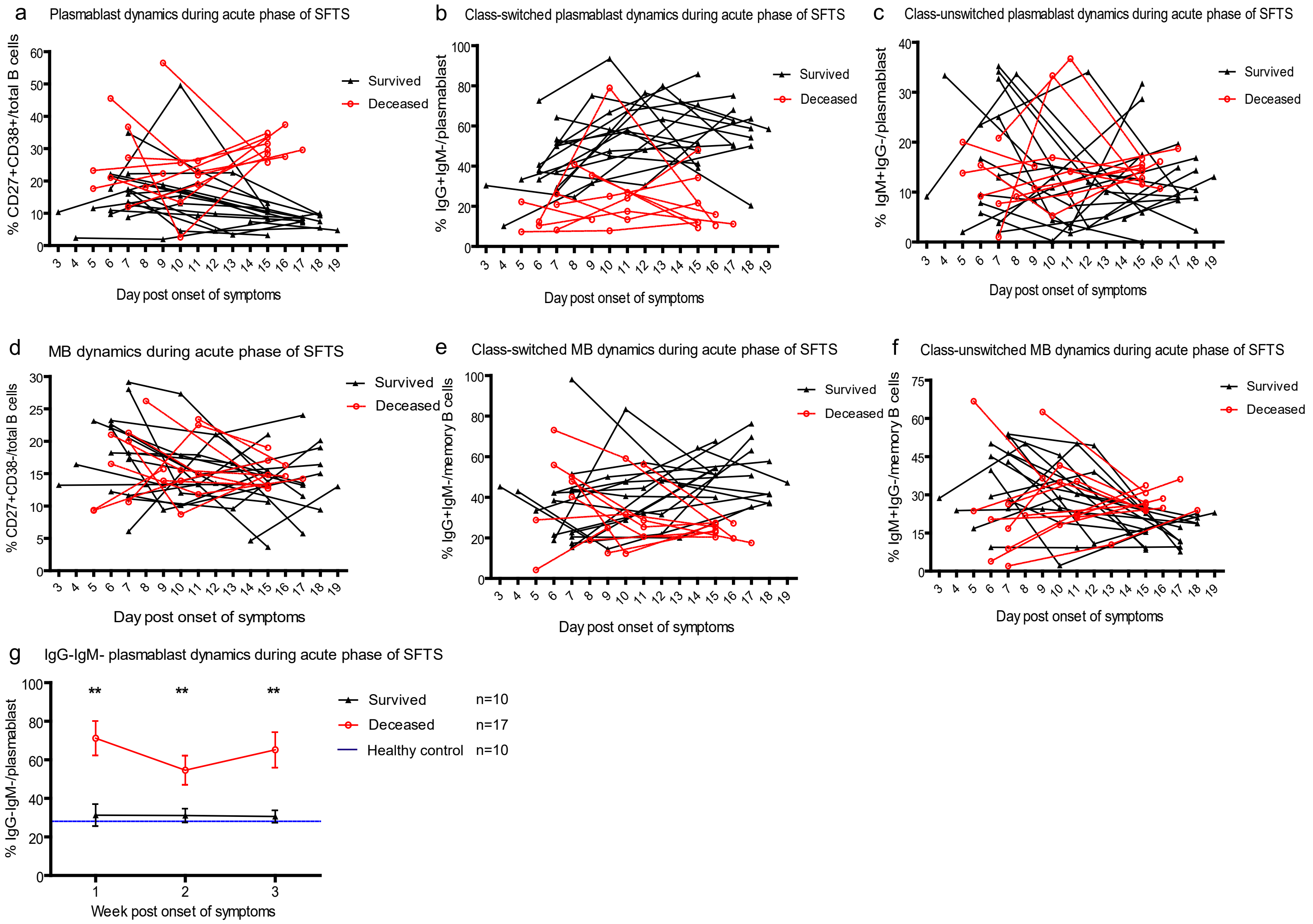

Supplementary Fig. 3 Dynamics of peripheral antibody-secreting cells of SFTS patients during acute phase. The percentage of peripheral plasmablasts (PB) and memory B cells (MB) as defined by CD38 and CD27 expression in 17 survived and 10 deceased patients over the entire three-week clinical course post symptom onset are shown in (a) and (d). The percentage of positive intracellular IgG (b, e) and IgM (c, f) staining of PBs (b, c) and MBs (e, f) are shown. Each line represents an individual patient. Kinetics of IgG-IgM- plasmablasts during acute phase of SFTS are presented in (g). The means of numbers of the 17 survived and 10 deceased patients was compared as the proportion of IgG-IgM- cells in total plasmablasts. Significant difference was calculated using one way ANOVA. ** $p < 0.005$. Error bar represents the standard deviation.

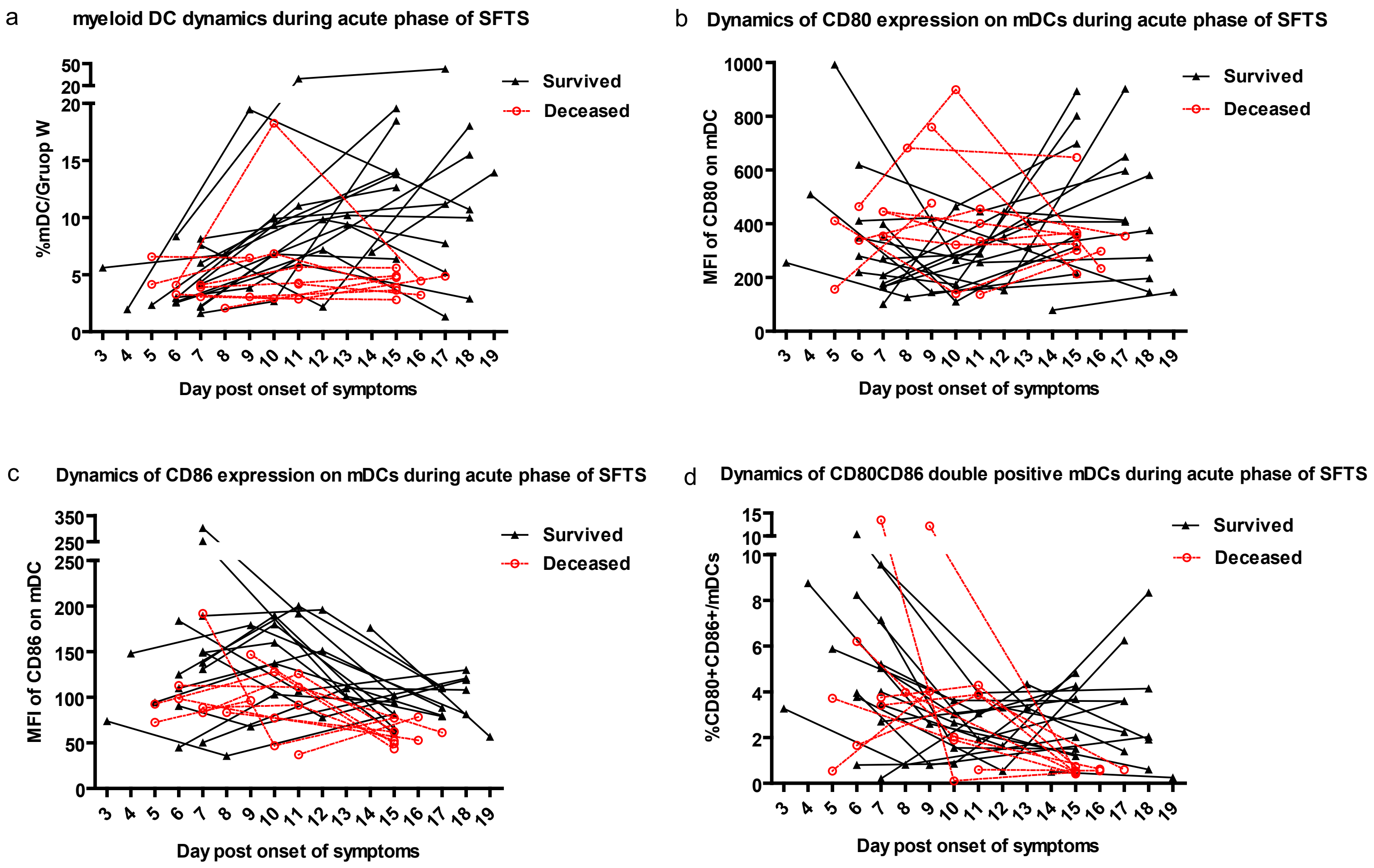

Supplementary Fig. 4 Dynamics of peripheral mDCs and the expression of co-stimulatory molecules during acute phase of SFTS. Peripheral mDCs population of 17 survived and 10 deceased patients over the entire three-week clinical course post symptom onset are shown in (a). MFIs of surface CD80 and CD86 of mDCs are shown in (b) and (c), respectively. The changes of percentage of CD80+CD86+ mDCs in the three-week clinical course are shown in (d).

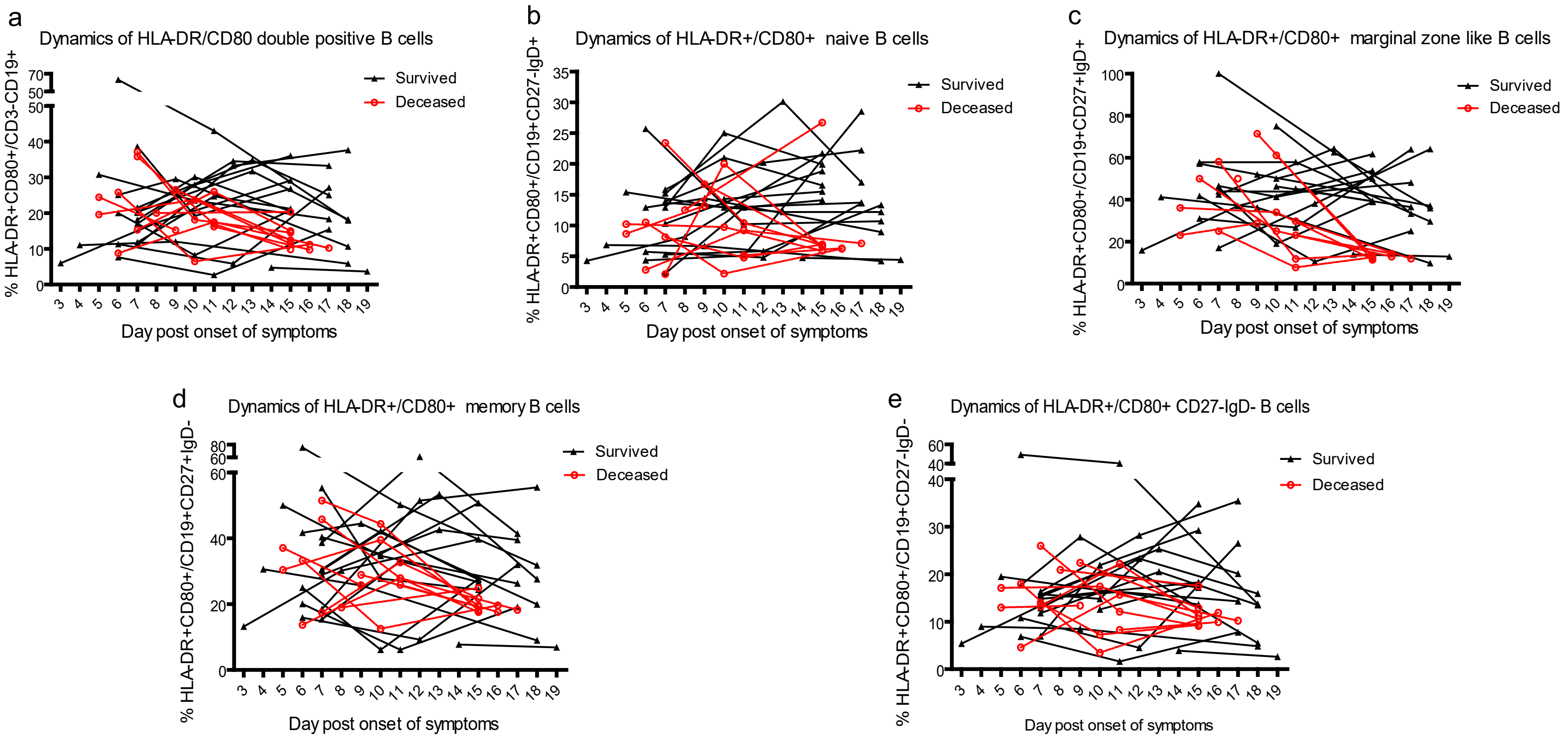

Supplementary Fig. 5 Dynamics of HLA-DR+CD80+ cells in peripheral B cell subsets during acute phase of SFTS. The percentage of HLA-DR+CD80+ cells as expressed in total B cells (a), naïve B cells (b), marginal zone like B cells (c), memory B cells (d) and CD27-IgD- B cells (e) in 17 survived and 10 deceased patients over the entire clinical course post symptom onset are shown. Each line represents an individual patient.

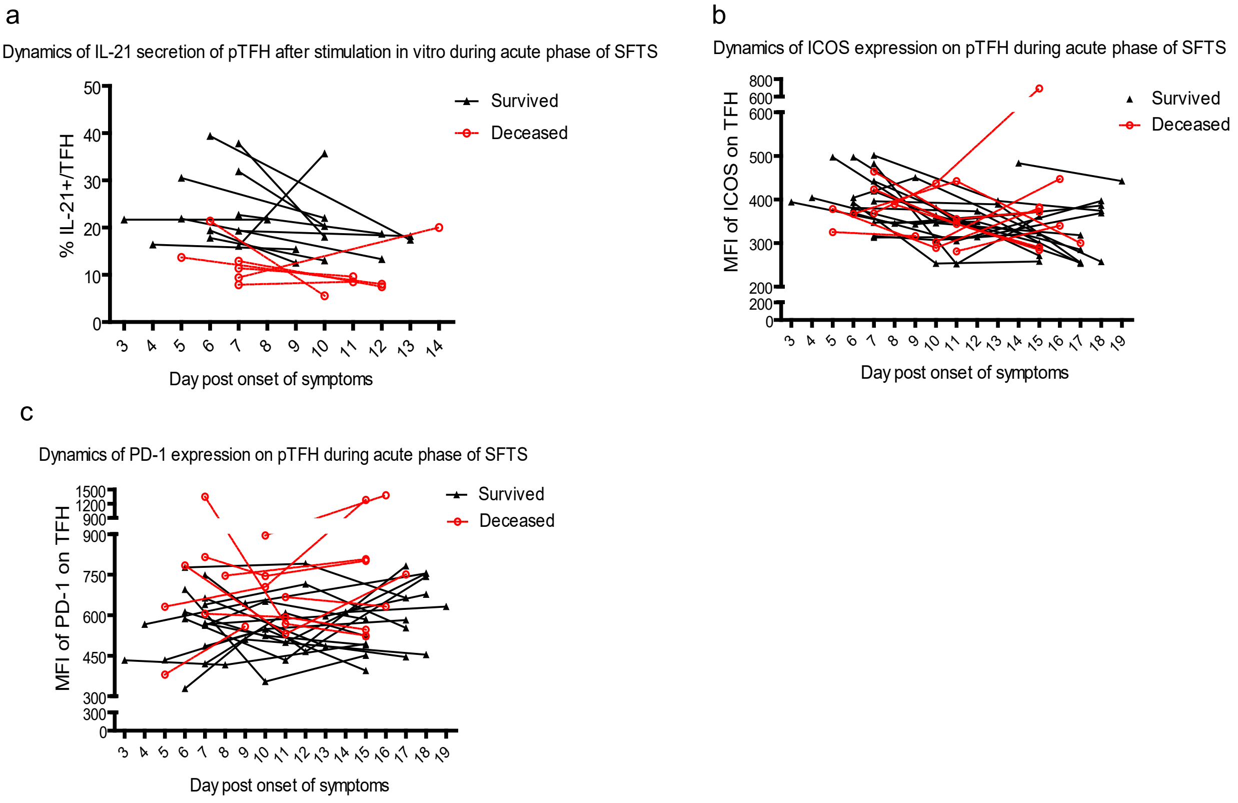

Supplementary Fig. 6 Dynamics of IL-21 positive pTfh cells and the expression of ICOS and PD-1 on pTfh during acute phase of SFTS. The percentage of IL-21 positive pTfh cells (a), MFI of ICOS (b) and PD-1 (c) on pTfh cells of 17 survived and 10 deceased patients over the entire clinical course or indicated dates post symptom onset are shown.
